# Facilitators and barriers for the implementation of intersectoral collaboration for health among Dutch primary and community care professionals: a qualitative study

**DOI:** 10.1136/bmjph-2026-004909

**Published:** 2026-11-06

**Authors:** Nils Keesmekers, Dennis De Ruijter, Ien Van De Goor, Willem Jan Jozef Assendelft, Stef Petrus Jacobus Kremers

**Affiliations:** 1 Department of Health Promotion, Maastricht University, Maastricht, The Netherlands; 2 Tranzo, Tilburg University, Tilburg, The Netherlands; 3 Primary Care and Community Care, Radboud University Nijmegen Medical Centre, Nijmegen, The Netherlands

**Keywords:** Public Health, Community Health, Preventive Medicine, Qualitative Research, Social Medicine

## Abstract

**Introduction:**

Healthcare systems increasingly emphasise collaborative, preventive and holistic care to address the rising burden of an ageing population, chronic diseases and workforce shortages. This study explores facilitators and barriers to the application of three inter-related practices in Dutch primary and community care, summarised as ‘intersectoral collaboration for health’: collaborative care, preventive care through lifestyle counselling and holistic approaches to care through the concept of Positive Health. The research question was: which factors facilitate or hinder intersectoral collaboration for health as experienced by primary and community care providers in the Netherlands?

**Methods:**

We conducted a qualitative study using semistructured interviews with 18 primary and community care providers involved in collaborative and preventive care in the Netherlands. Data were analysed using a quasi-inductive thematic approach, combining inductive identification of themes with deductive comparison to existing frameworks. Facilitators and barriers were categorised as either individual or socio-environmental factors.

**Results:**

Across all three practices, perceived benefits and intrinsic motivation facilitated application while limited knowledge, time and resources as well as limited interaction acted as barriers. Collaboration was facilitated by mutual trust and understanding between care providers, whereas lifestyle counselling depended on providers’ perceptions of their professional roles and motivation in care recipients. The application of Positive Health emerged as a facilitator for both collaboration and lifestyle counselling but sometimes competed with other approaches.

**Conclusions:**

Findings indicate that facilitators and barriers to intersectoral collaboration for health occur at multiple levels. As such, its application requires coordinated efforts. Application of Positive Health as a shared language and stable funding of collaborative and preventive care contribute substantially to sustainable change. Education and training can strengthen professionals’ attitudes and competencies for collaborative and preventive care.

**WHAT IS ALREADY KNOWN ON THIS TOPIC**

- Healthcare systems are under growing pressure due to increasing care demands and workforce shortages.
- Collaborative and preventive care are promoted as key strategies to ensure sustainable care delivery.
- In the Netherlands, the Positive Health framework supports these efforts by fostering collaboration and lifestyle counselling in primary and community care.

**WHAT THIS STUDY ADDS**

- Collaboration, preventive lifestyle counselling and the Positive Health framework function as mutually reinforcing components of intersectoral collaboration for health.
- Care providers recognise the importance of intersectoral collaboration for health, but face structural limits that hinder implementation.
- Positive Health offers a shared language that can bridge sectors, though its use depends on sufficient familiarity with the framework.

**HOW THIS STUDY MIGHT AFFECT RESEARCH, PRACTICE OR POLICY**

- For research, this study provides a basis for developing and testing strategies that strengthen intersectoral collaboration for health.
- For practice, this study further supports the need for interprofessional learning to build shared language, skills and knowledge for collaborative and preventive care.
- For policy, this study stresses the need for improved role alignment and sustainable funding for collaborative and preventive care.

## Introduction

 Healthcare systems worldwide are facing increasing pressure on affordability, accessibility and quality of care. Demand for healthcare is growing due to factors such as care recipients’ expectations,^1^ an ageing population and the increased prevalence of chronic illness and multimorbidity.^2,3^ Globally, the burden of disease from non-communicable diseases rose from approximately 1.45 billion disability-adjusted life years (DALYs) in 2010 to 1.80 billion DALYs in 2023, with coronary artery disease, stroke and diabetes as the leading contributors.^4^ At the same time, healthcare staff and resources around the world are becoming increasingly scarce due to declining workforces and budget cuts.^3,5,6^

Driven by the need for more efficient and affordable healthcare, promoting health and preventing illness are increasingly common strategies to reduce healthcare expenses.^7,8^ The adjustment of lifestyle behaviours, for example through lifestyle counselling, represents an essential form of preventive care^9^ which is linked to significant reductions in the incidence and progression of chronic illness.^10,11^ Healthcare professionals, in particular primary care providers (PCPs) and community care providers (CCPs), play a key role in lifestyle counselling. As a common point of entry in healthcare, PCPs and CCPs do not only coordinate care and identify opportunities for lifestyle adjustment, but also inform and counsel individuals on lifestyle behaviours.^12,13^ However, PCPs and CCPs cannot do this alone. Effective lifestyle counselling requires coordination and collaboration across organisations, disciplines and sectors.^11,14,15^ For example, Jennings and Astin^16^ highlight that preventing cardiovascular diseases involves not only medical interventions by physicians, but also lifestyle and behavioural counselling by non-medical disciplines.

Alongside an increased emphasis on prevention and lifestyle, more recent approaches to health and care advocate for holistic perspectives on health that consider psychological and social dimensions in addition to the traditional focus on physical functioning.^17,18^ Such perspectives are closely aligned with person-centred care, in which care is organised around individuals’ needs and their environment rather than around individual diseases or professional domains.^18,19^ Holistic perspectives may uncover health needs that extend beyond the expertise and responsibilities of individual care professionals and disciplines, further underlining the importance of collaboration. Additionally, it is argued that holistic approaches can support effective integration of lifestyle medicine, given that lifestyle behaviours are shaped by individual, social and environmental factors.^20^

Despite broad recognition of the importance of collaboration and lifestyle counselling^14,21–23^ these strategies remain underused in daily practice. Similar implementation gaps have been reported for holistic approaches to health, which may be widely endorsed but not consistently translated into routine practice.^24,25^ PCPs and CCPs report that they often forego or postpone lifestyle counselling, for example, due to limited knowledge, skills and time.^26–28^ Furthermore, care is often fragmented across different systems and providers^29^ meaning that duplicate or even conflicting services may be provided by different care providers due to a lack of coordination.^30^ Even though care providers generally believe collaboration to be beneficial, limited knowledge and uncertainty regarding roles and responsibilities, among others, hinder care providers in effective collaboration.^31,32^ For holistic approaches, variations in terminology and conceptualisations might complicate their consistent operationalisation in practice.^33,34^

Against this background, and given the interdependencies between collaborative, preventive and holistic approaches to care, we propose intersectoral collaboration for health as an overarching concept that describes collaboration between care providers across sectoral boundaries, oriented towards promoting and maintaining health. A holistic perspective on health provides an important conceptual foundation for both collaborative and preventive care. For collaborative care, recognising that health is shaped by interconnected physical, psychological and social dimensions highlights that care needs may extend beyond the expertise or responsibilities of individual professionals or sectors. For preventive care, a holistic perspective draws attention to the interplay between individual, social and environmental factors and recognising opportunities to promote health.

The distinction between primary and community care is particularly relevant in the Netherlands, where these sectors operate within different organisational, professional and financing structures. Primary care includes professionals such as dieticians, physiotherapists and general practitioners, with the latter as the first point of contact for many healthcare needs and as gatekeepers to more specialised medical care.^35^ Much of curative care, including general practice and other primary care services, is financed through compulsory health insurance.^35^ Community care, including social support, is provided by social workers and welfare organisations, among others and is largely organised and funded through municipalities.^36^ While care included in health insurance is based on a nationally defined standard package,^35^ social care is decentralised to municipalities, who have considerable autonomy in how services are organised within their responsibilities and resources.^36^ Although these sectors serve overlapping populations and frequently encounter inter-related health and social needs, collaboration may be complicated due to differences in responsibilities, financing structures and terminology.^37^

Within the Dutch context, Positive Health has become a prominent framework to operationalise a holistic perspective on health. Positive Health considers health as ‘the ability to adapt and self-manage in light of social, physical, and emotional challenges’.^38^ The framework encourages care providers to consider multiple dimensions of health as well as individuals’ broader circumstances rather than focusing primarily on disease or physical functioning. In practice, Positive Health also encourages collaboration between professionals when needs extend beyond their own expertise.^39^ Since its conception in 2011, Positive Health has become increasingly integrated in Dutch policy and practice for healthcare and welfare,^40,41^ and it is currently also gaining traction internationally.^42–44^ Its notable prominence in Dutch policy and practice makes Positive Health a particularly relevant framework for examining how a holistic perspective can relate to collaborative and preventive care within intersectoral collaboration for health.

While recent research into healthcare professionals’ experiences with Positive Health has highlighted its potential to serve as a shared framework that supports collaboration,^34,45,46^ research on facilitators and barriers for collaboration, lifestyle counselling and holistic approaches has mostly examined these practices separately.^14,20,28,31,32,45,47^ Consequently, little is known about how the determinants of these inter-related practices overlap or differ for care providers, and what this means for the implementation of intersectoral collaboration. The present study therefore aims to explore factors that facilitate or hinder intersectoral collaboration for health as experienced by PCPs and CCPs in the Netherlands. Specifically, we examine collaboration between primary and CCPs as its intersectoral dimension, lifestyle counselling as an example of its preventive orientation and Positive Health as a prominent operationalisation of a holistic perspective in the Netherlands.

## Methods

Given the exploratory nature of the research question, we conducted a qualitative study to explore PCPs’ and CCPs’ experiences implementing intersectoral collaboration for health in daily practice. Reporting follows the Consolidated Criteria for Reporting Qualitative Research (COREQ).^48^ The completed SRQR checklist is provided in online supplemental appendix 1.

### Recruitment

Individuals were eligible if they worked as PCPs (eg, dieticians, general practitioners, nurse practitioners, midwives, physical therapists) and CCPs (social workers) in the geographical scope of the research consortium (ie, the provinces of Limburg, Gelderland, Noord-Brabant and Zeeland, the Netherlands).

Participants were mainly recruited through snowball sampling at health and social work organisations as well as educational institutes with healthcare-related programmes. Between November 2022 and February 2023, 14 representatives from organisations that were part of our research consortium were prompted to invite PCPs and CCPs, and to extend the invitation in their network. Additionally, participants were recruited through a newsletter of Zorgbelang Brabant-Zeeland, an organisation advocating for the improvement of health and social care with connections to patient organisations, municipalities, (health)care providers and healthcare insurance companies. Approximately 12 participants were initially anticipated. Additional participants were recruited to account for cancellations; all 18 recruited participants completed an interview. Recruitment was then discontinued as the final four interviews did not yield meaningful new themes.

Interested PCPs and CCPs contacted the main researcher (NK) via email or telephone and received information about the study purpose, interview procedure, time investment, data security, right to withdraw and absence of compensation. Interviews were scheduled after participants agreed to these terms.

The researchers had no prior relationship with the participants before the study commenced. Participants were informed that the research aimed to identify facilitators and barriers for the application of collaboration, lifestyle counselling and Positive Health to inform the development of educational material for healthcare providers.

### Patient and public involvement

Patients or members of the public were not involved in the development of the research question, study design, recruitment, conduct or analysis of this study. The study focused on the experiences of PCPs and CCPs. Following completion of the study, all participants received a summary of the findings and were invited to provide feedback on their accuracy and interpretation.

### Procedure

Interviews with individual participants were conducted between November 2022 and February 2023. Interviews lasted approximately 1 hour and were conducted in an online setting, using Microsoft Teams videoconferencing software. Interviews were always preceded by written informed consent. A semistructured interview guide (online supplemental appendix 2) was used to provide consistency across interviews and minimise interviewer bias, such as asking leading questions or selectively probing based on prior assumptions. Following consent, all interviews were recorded with a digital voice recorder, transcribed naturalistically^49^ and pseudonymised by replacing identifying information with participant identifiers.

### Materials

The research team developed and iteratively refined a semistructured interview guide through internal discussions. The guide was pilot tested with a physical therapist, recruited through convenience sampling, to assess question clarity and relevance. No substantive changes were required. The final interview guide was based on the three topics of interest: collaboration, lifestyle counselling and Positive Health. It contained open-ended questions and follow-up probes. For each theme, participants were first asked about their familiarity and experiences, followed by questions that explored perceived facilitators and barriers to application in their daily work. An example of a question used is: ‘Which reasons do you see to collaborate with other care providers in your work?’ The full interview guide can be found in online supplemental appendix 2.

### Data analysis

Data were analysed in a quasi-inductive manner informed by grounded theory.^50^ This approach allowed themes to emerge from the data while still enabling comparison with existing theories,^50^ fitting with the exploratory aim of this study. Following familiarisation through transcription of interview recordings and reading transcripts, initial codes were generated inductively through open coding. Next, coded data were grouped deductively into individual and environmental (socio-structural) categories, reflecting a common distinction in behavioural research.^51–53^ Examples of individual factors are attitude, beliefs, knowledge, emotions, skills and habits. Environmental factors include social support, regulations as well as other general social and environmental conditions in which people work.^52^ Theme development was based on the complete dataset, while illustrative quotations were selected for their clarity and relevance to the identified themes.

ATLAS.ti V.23.2.0 was used for data processing and calculation of inter-rater reliability. An initial coding scheme was developed, after which four transcripts were independently double-coded by NK and DDR. After the first two transcripts, discrepancies were discussed and the coding scheme was refined. A third transcript yielded a Krippendorff’s Alpha coefficient^54^ of 0.85. All remaining transcripts were coded by NK. Following completion of coding, one randomly selected transcript was independently coded by DDR to assess coding consistency, yielding a Krippendorff’s Alpha coefficient of 0.94.

## Results

### Participant characteristics

Interviews were held with 18 PCPs and CCPs, of which 14 were female and 4 male. Their ages ranged from 26 to 72 years with a mean age of 43.3 (SD=13.3). The sample included general practitioners (n=4), social workers (n=2), dieticians (n=2), physical therapists (n=2), lifestyle coaches (n=2), a well-being coach, a nurse practitioner, a speech therapist, a nurse specialist youth healthcare, a youth public health physician and a behavioural expert working for a municipality. Participants worked in the provinces of Brabant (n=6), Limburg (n=5), Gelderland (n=4) and Zeeland (n=3).

#### Theme 1: collaboration between care providers

##### Facilitators

With regard to individual facilitators, participants considered collaboration as a way of working that provides them with benefits. These benefits mostly related to solving specific health problems that PCPs and CCPs encounter.

The solution to problems we encounter during consultation hours often does not lie in our own domain, but more often in poverty, loneliness, addiction – things like that, where we do need help from others (Participant 6, general practitioner).

Participants also described knowledge about and trust in collaboration partners as an important facilitator for collaboration. This relates to knowing others on a personal level (by name and face) and on a professional level (what others do and where others’ expertise lies), as well as knowing how others’ expertise can complement theirs.

As for socio-environmental factors, interaction between PCPs and CCPs was described as an influential facilitator for collaboration. Participants frequently mentioned that collaboration benefited from mutual agreements and efforts to speak each other’s (professional) language. The language of Positive Health was regularly mentioned in this context, for example, by Participant 10 (well-being coach): “It’s nice if you have a similar language, and that you can also use that language with your client or patient and that it is also clear for them that [care providers] speak the same language*.*” Informal interactions were also reported to be beneficial for collaboration, “because the more you like someone, the more you are willing to do for them” (Participant 1, social worker).

Furthermore, participants explained that collaboration was heavily influenced by the physical or digital work environment. For example, participant 2 (dietician) explained, “We also sit in with some physical therapists where we provide consultations, and it’s really easy because you can talk to them for example, during the lunch break.” Digital tools used for communication between care providers were described to similarly facilitate collaboration:

We have a system that allows us to see medical data, so we can already see what they did […] and inside this system we also have a secure way to send a quick message, to provide an update about what we did […] which makes for an easy and accessible way to be connected (Participant 2, dietician).

##### Barriers

Limited knowledge regarding collaboration partners’ professions was a recurring theme, suggesting an important individual barrier for collaboration. This lack of knowledge also includes misconceptions regarding roles and responsibilities:

We blame each other because we don’t know each other’s domain very well. […] What mental health services do is say ‘yes, but school has to do it.’ And then the school says, ‘yes, but we are not allowed to pay for healthcare with education funds, so healthcare has to…’ and then a whole story follows there. Accusations are made and I think that’s because […] they just don’t know what’s going on (Participant 7, behavioural expert).

Less frequently mentioned barriers to collaboration were concerns regarding care recipients’ privacy and perceived competition with other care providers. One participant described perceived cultural differences between disciplines as a potential barrier:

I always say: sportspeople are doers but social workers, for example, they are talkers. And that’s a big cultural difference that sometimes is reflected in the organisation (Participant 10, well-being coach).

Limited interaction and high turnover among care providers posed important socio-environmental barriers to collaboration. Participant 2 (dietician) mentioned that “with professionals who you don’t see very often, it takes a lot of time and energy to regularly get in touch.” Similarly, Participant 6 (general practitioner) noted that “it is a coming and going of care providers. As a referrer, you cannot build a collaborative relationship with them.” In addition, time and financing were often described as influential factors that can hinder collaboration, for example, by Participant 7 (behavioural scientist): “Everyone is incredibly busy, so if you make time for [networking], it’s at the expense of your core business, so it’s time and money.” Regarding funding for collaboration within primary care, Participant 6 (general practitioner) reported that “when the funding was lost, so was any enthusiasm to continue with any kind of initiative.”

### Theme 2: lifestyle counselling

#### Facilitators

Participants often mentioned positive outcome expectations relating to lifestyle counselling, which was interpreted as an important individual facilitator for its application. Participant 3 (physical therapist) said: “for COPD, we know that if they come to exercise twice a week, that can prevent one hospitalisation every year.” Additionally, the expectation that lifestyle counselling required little time investment also facilitated its application: “It helps to plant that seed […] and that actually takes less than a minute” (Participant 14, general practitioner). Some participants also mentioned that the application of lifestyle counselling, and prevention more generally, was facilitated by individual characteristics or intrinsic motivation: *“*I believe that […] prevention is something you breathe, or something. It is not something you should be told to do, but you really need to feel and see its importance” (Participant 7, behavioural scientist).

Socio-environmental facilitators that were mentioned included agreements on the use of structured approaches and frameworks such as Motivational Interviewing or Positive Health. Care providers described that these approaches provide them with specific tools to guide lifestyle counselling: “And I can say, you need to drink less alcohol, but maybe that person is thinking about smoking less […] so that’s where we use Positive Health as well” (Participant 5, lifestyle coach). At the same time, participants mentioned regular interaction between care providers and care recipients as a facilitator:

The GP sees their clients once a year, and the other contact takes place with nurse practitioners. So they know them better, see them more often […] and they also make an effort for lifestyle counselling (Participant 2, dietician).

#### Barriers

On the individual level, participants from various disciplines mentioned doubts about whether they should consider lifestyle counselling part of their work. For example, a general practitioner mentioned: “We do indicated prevention, so when you’re already ill, you have diabetes, that belongs to us. But for primary […] prevention, it is said, ‘we don’t know if that’s our job’” (Participant 13, general practitioner). Although this participant referred to indicated prevention, their description more closely resembles care-related prevention, which further illustrates ambiguity regarding preventive roles and responsibilities. Similarly, a physical therapist said: “The question is, are we as physical therapists well-equipped for prevention, aren’t other disciplines already better at this?” (Participant 17, physical therapist).

On the socio-environmental level, participants reported that specific characteristics of care recipients could hinder lifestyle counselling: “Especially the older generation, there I notice the difference that they […] come to you and you need to fix it for them, whereas prevention and lifestyle is considered from a different perspective” (Participant 11, lifestyle coach). Furthermore, time and funding pose a considerable barrier as described by a general practitioner: “So you have little time and opportunity to (create) a treatment relationship, whereas a lot of what we do is […] convincing and motivating people to […] healthier behaviour, but you barely get the time and that time is compensated very poorly” (Participant 6, general practitioner).

### Theme 3: Positive Health

#### Facilitators

Individual facilitators described by participants for the application of Positive Health were positive outcome expectations and personal characteristics, often related to intrinsic motivation or alignment with their own values. Participant 6 (general practitioner) mentioned: “I do a lot of things from intrinsic motivation, and I think that applies for a lot of GPs.” This sentiment was reported by participants from other backgrounds, including dieticians, social workers and the behavioural scientist.

Socio-environmental facilitators described by participants included the use of protocols as well as other holistic practices similar to Positive Health:

We often do not call it by name, but the main idea is the same, and we do it by using other methods, […] everything with the purpose to have a broad view on health and people’s own capabilities (Participant 7, behavioural expert).

Similarly, the availability of time for more in-depth conversations and the content of care requests can play a facilitating role, as described by a physical therapist who said, “it depends on if it is […] a local problem or if it influences multiple areas of [a patient’s] life” (Participant 3, physical therapist). A dietician explicitly connected Positive Health and lifestyle counselling in this regard:

Very often, people come to us and say, ‘I want to lose weight,’ ‘my cholesterol needs to come down’ […] and if you continue the conversation, you end up with mental well-being, quality of life, daily functioning […] as the end goal that they want to improve on. So very often, what they want to improve is a way to improve another dimension [of Positive Health] (Participant 2, dietician).

#### Barriers

On the individual level, negative outcome expectations and limited knowledge among care providers regarding Positive Health were commonly reported individual barriers hindering its application. Socio-environmental barriers related to the content of care requests and care recipient characteristics:

If [a care recipient] really says, you know, […] I don’t want to talk about all of that, you should accept that (Participant 3, physical therapist).

Some participants mentioned that the use of similar holistic practices could also pose a barrier. This was described by a general practitioner who mentioned: “I think I don’t apply Positive Health […] because I use the SCEGS-method […] and I see less need to use a different tool” (Participant 14, general practitioner). The SCEGS-method refers to an interview structure that explores Somatic, Cognitive, Emotional, Behavioural and Social influences for care recipients’ problems.^55^

## Discussion

This study identified a variety of individual and environmental factors that influence the implementation of intersectoral collaboration for health in primary care and community care settings. For collaboration, lifestyle counselling and holistic approaches such as Positive Health, application is facilitated by perceived benefits and intrinsic motivation in care providers. Barriers for application of these three practices are a lack of knowledge, time and resources in care providers, as well as limited interaction with care providers or care recipients. The practice of Positive Health itself was mentioned to facilitate both collaboration and lifestyle counselling, further strengthening the notion that these practices are related. Collaboration is facilitated by understanding of and trust in other care providers, and hindered by high turnover and privacy concerns. Lifestyle counselling is facilitated or hindered by perceived role identity, perceived time investment and care recipient characteristics. Applying Positive Health in daily practice can be facilitated or hindered by both the content of care requests as well as the use of similar practices.

These findings extend existing literature on collaborative and preventive practices in healthcare. For collaboration, our findings underscore that knowledge and trust regarding collaboration partners is essential. This supports earlier work that identified trust, role clarity and (in)formal communication as key determinants of successful collaboration.^31,32^ Our results additionally suggest that these influences can be strengthened with the use of a common language such as Positive Health. For lifestyle counselling, our results underscore the importance of knowledge, role clarity and outcome expectations as determinants for counselling behaviour.^28,56^ The influence of care recipient characteristics, such as motivation or readiness to change, has also been observed in previous research.^27^ Our findings build on this evidence by showing that lifestyle counselling is supported by collaboration with other care providers and the use of holistic approaches such as Positive Health. Together, these results highlight the value of collaboration for lifestyle counselling and the importance of communication between care providers to ensure knowledge, trust and role clarity.

The findings also highlight the potential of holistic approaches in supporting collaboration and lifestyle counselling. Recent research has been published that describes the potential of Positive Health as a shared language supporting collaboration.^34,45^ Our findings additionally illustrate the value of Positive Health for lifestyle counselling and provide insight on how its perceived compatibility with existing methods can both facilitate and hinder application. In summary, these results suggest that holistic approaches such as Positive Health may serve as a supportive framework for intersectoral collaboration for health.

Taken together, the present findings also reinforce our introduction of intersectoral collaboration for health as an approach that integrates a broad view on health while centring care around the individual and their environment. Our results specifically illustrate that collaborative care, preventive care and a holistic approach are not isolated practices, but should be considered as mutually reinforcing components of intersectoral collaboration for health.

### Implications

The combination of individual and socio-environmental factors that influence the implementation of intersectoral collaboration for health suggests that opportunities to improve it can be found across multiple socio-ecological levels.^57^ With the potential impact of Positive Health as a universal language for collaborative and preventive efforts, its application in (health)care should be encouraged through policies, organisations and individuals.

On the policy level, the application of collaboration, lifestyle counselling and Positive Health requires consistent policy that connects these three practices as intersectoral collaboration for health. In the Netherlands, this is partly realised, for example, through the Integral Care Agreement and the Supplementary Health and Welfare Agreement.^41^ However, our participants reported that funding for collaboration and prevention is unstable and that roles for preventive care are unclear. Intersectoral and preventive care should be funded in a structural manner so that it is no longer dependent on temporary funding. Moreover, responsibilities for preventive care should be clarified to address ambiguity in professional roles and to support shared ownership of preventive tasks. On the national level, this includes that professional associations (eg, for general practitioners) further shape role expectations and guidelines on prevention and lifestyle. On the regional level, intersectoral collaboration networks can facilitate the conditions for these responsibilities in local practice.

On the organisational level, continuity of care and interaction among care providers and with care recipients is essential for intersectoral collaboration for health. As high staff turnover and fragmented communication channels undermine collaborative relationships,^58,59^ organisations should invest in stable teams, shared communication systems and opportunities for informal contact across disciplines and sectors. Leadership can support these conditions by fostering a shared vision, clarifying roles and responsibilities and promoting communication across professional and sectoral boundaries.^60,61^ Integrating the practice of holistic approaches such as Positive Health in (health)care organisations may further help to facilitate communication among care providers, using its potential of a shared language that provides a shared vision and facilitates mutual understanding.^34^

On the individual level, the role of intrinsic motivation and perceived relevance for care providers’ behaviour indicates that trainings or other interventions should extend beyond technical skill-building and also focus on values and perceived benefits. Interprofessional education may help care providers to recognise and coordinate their role in collaborative and preventive care,^62^ and integrate collaboration and lifestyle counselling through a shared language such as Positive Health.

### Strengths and limitations

A key strength of this study is that it provides insights from a diverse sample of professionals across primary care and community care settings. This sample allows for the identification of facilitators and barriers in a broad range of care contexts. The focus on collaboration, lifestyle counselling and Positive Health as inter-related practices also allows for the identification of shared as well as unique facilitators and barriers. In addition, the inclusion of Positive Health as operationalisation of a holistic approach is particularly valuable given that these approaches are increasingly embraced, yet organisations struggle with its application in practice.^34^

Nevertheless, some limitations should be considered when interpreting the results of this study. First, because this study operationalised a holistic perspective primarily through Positive Health, some identified facilitators and barriers may also apply to other holistic or person-centred approaches. Furthermore, recruitment partly relied on existing networks and collaborations within the research consortium; as such, participants may have been more familiar with or motivated towards Positive Health and collaboration than the average Dutch care provider. Still, participants described a broad range of experiences including mentions of other, less experienced care providers and some participants specifically reported to not apply Positive Health in specific situations. Although a wide range of professionals was represented, not all disciplines active in primary and community care were included. Hence, it is possible that additional interviews with under-represented disciplines such as dental professionals and occupational therapists may reveal further nuances. Finally, as with all qualitative research, findings reflect the experiences and contexts of participating care providers as well as the researcher’s interpretations. To enhance credibility, data were coded independently by two researchers and discussed iteratively until consensus was reached. The main researcher (NK), trained as a health and behavioural scientist, was familiar with primary care but not previously involved with the concepts of (intersectoral) collaboration, lifestyle counselling or Positive Health. This position allowed for an open and unbiased perspective towards these practices, while regular discussions with the research team helped to check interpretations and strengthen the credibility of the findings.

### Recommendations for future research

Building on insights from the present study, research could examine how targeted strategies could be developed and tested to support intersectoral collaboration for health across primary care and community care. Such strategies should focus on training or structural and sustained incentives for collaboration. Their impact on (health)care practice should be evaluated in terms of short-term learning or behavioural outcomes (eg, changes in role clarity, collaboration or use of holistic approaches during consultations) as well as longer-term outcomes (eg, continuity of collaborative and preventive initiatives or referral patterns across primary and community care).

Moreover, longitudinal implementation studies could further investigate how funding and policies influence the continuity of collaborative and preventive initiatives. For example, studies could evaluate which funding structures promote sustainable collaborative efforts. Additionally, future research should explore facilitators and barriers in under-represented contexts. These contexts include other countries adopting Positive Health (eg, Belgium, Germany, Columbia, Iceland and Japan)^45^ and disciplines not well represented in this study (eg, dental professionals and occupational therapists).

## Conclusions

This study contributes to a growing understanding of how collaborative care, preventive care and holistic approaches are inter-related and mutually reinforcing components of intersectoral collaboration for health. Its findings highlight that while many care providers recognise the value of collaborative and preventive care, application is often constrained by limited time, unstable funding, unclear role definitions and fragmented communication across sectors. Addressing these challenges requires alignment across multiple levels, from policy and organisational levels to individual care providers. The individual and socio-environmental determinants in this study offer a foundation for designing future interventions that strengthen intersectoral collaboration for health in primary and community care settings.

## Supplementary material

10.1136/bmjph-2026-004909 online supplemental appendix 1

10.1136/bmjph-2026-004909 online supplemental appendix 2

## Data Availability

Data are available upon reasonable request.
