# Supplementary material for: Facilitators and barriers for the implementation of intersectoral collaboration for health among Dutch primary and community care professionals: a qualitative study
Source: BMJ Public Health. 2026 Nov 6;4(4):e004909. doi: 10.1136/bmjph-2026-004909 (PMC13756415; doi:10.1136/bmjph-2026-004909)
Supplement: Supplementary file 1 [file bmjph-4-4-s001.pdf]

The SRQR reporting checklist

For checking that qualitative health research articles can be understood and used by everyone

Note

If you have not used a reporting guideline before, read about how and why to use them and check whether SRQR is the most applicable reporting guideline for your work.

Reporting guidelines are most useful when used early in research. When writing a manuscript or application, consider using the Full Guidance where you'll see explanations and examples for each item.

After writing, demonstrate adherence by completing this checklist:

1. Specify where each item is described (see Note 1).
2. Cite this checklist (See Note 2).
3. Include your completed checklist as a supplement when submitting to a journal so that future readers can use it to find information.

	Item Description	Location (or reason for not reporting)
**Title & Abstract**		
Title	Describe the nature and topic of the study. Identify the study as qualitative or indicate the approach or data collection methods.	Title Page
Abstract	Summarise the key elements of the study using the abstract format of the intended publication.	Abstract
**Introduction**		
Problem Formulation	Describe the problem/phenomenon studied, its significance, relevant theory and empirical work, and gaps in current knowledge.	Introduction; paragraphs 1–8
Purpose or research question	Describe the purpose of the study and specific objectives or questions.	Abstract<br>Introduction; paragraph 8
**Methods**		
Qualitative approach and research paradigm	Describe your qualitative approach, your guiding theory (if appropriate), and research paradigm, and reasons for your choices.	Methods; paragraph 1<br>Methods; Data Analysis; paragraph 1

Researcher characteristics and reflexivity	Describe how researchers' characteristics may influence the research, including personal attributes, qualifications/experience, relationship with participants, assumptions, and/or presuppositions; potential or actual interaction between researchers' characteristics and the research questions, approach, methods, results and/or transferability.	Methods; Recruitment; paragraph 4<br>Discussion; Strengths and limitations; paragraph 2
Context	Describe the setting/site(s) in which the study was conducted, why it was selected, and any other salient contextual factors that may influence the study.	Introduction; paragraphs 6 & 7<br>Methods; Recruitment; paragraphs 1 & 2
Sampling strategy	Describe how and why research participants, documents, or events were selected; criteria for deciding when no further sampling was necessary, and the rationale for those criteria.	Methods; Recruitment; paragraphs 1 & 2
Ethical issues pertaining to human subjects	Describe any approval by an appropriate ethics review board and participant consent, or explain any lack thereof. Describe any other confidentiality and data security issues.	Methods; paragraph 1<br>Methods; Recruitment; paragraph 3<br>Methods; Procedure; paragraph 1
Data collection methods	Describe the types of data collected; details of data collection procedures including (as appropriate) start and stop dates of data collection and analysis, iterative process, triangulation of sources/methods, and modification of procedures in response to evolving study findings. Describe your rationale for these choices.	Methods; Procedure; paragraph 1<br>Methods; Materials; paragraph 1
Data collection instruments and technologies	Describe any instruments (e.g., interview guides, questionnaires) and devices (e.g., audio recorders) used for data collection; describe if/how the instrument(s) changed over the course of the study.	Methods; Procedure; paragraph 1<br>Methods; Materials; paragraph 1<br>Appendix 1
Units of study	Describe the number and relevant characteristics of participants, documents, or events included in the study. Describe the level of participation.	Methods; Recruitment; paragraphs 1 & 2<br>Results; Participant Characteristics; paragraph 1
Data processing	Describe the methods for processing data prior to and during analysis, including transcription, data entry, data management and security, verification of data integrity, data coding, and anonymisation / deidentification of excerpts.	Methods; Recruitment; paragraph 3<br>Methods; Procedure; paragraph 1<br>Methods; Data Analysis; paragraphs 1 & 2

Data analysis	Describe the process by which inferences, themes, etc. were identified and developed, including the researchers involved in data analysis; usually references a specific paradigm or approach. Describe why you chose this process.	Methods; Data Analysis; paragraphs 1 & 2
Techniques to enhance trustworthiness	Describe any techniques to enhance trustworthiness and credibility of data analysis,(e.g., member checking, triangulation, audit trail). Describe why you chose these techniques.	Methods; Patient and Public Involvement; paragraph 1<br>Methods; Materials; paragraph 1<br>Methods; Data Analysis; paragraph 2<br>Discussion; Strengths and limitations; paragraph 2
**Results**		
Synthesis and interpretation	Describe the main findings (e.g., interpretations, inferences, and themes); might include development of a theory or model, or integration with prior research or theory.	Results; Theme 1: Collaboration between care providers; paragraphs 1–11<br>Results; Theme 2: Lifestyle Counselling; paragraphs 1–5<br>Results; Theme 3: Positive Health; paragraphs 1–8
Links to empirical data	Provide evidence (e.g., quotes, field notes, text excerpts, photographs) to substantiate analytic findings.	Results; Theme 1: Collaboration between care providers; paragraphs 1–11<br>Results; Theme 2: Lifestyle Counselling; paragraphs 1–5<br>Results; Theme 3: Positive Health; paragraphs 1–8
**Discussion**		
Integration with prior work, implications, transferability, and contribution(s) to the field	Summarize the main findings, explain how findings and conclusions connect to, support, elaborate on, or challenge conclusions of earlier scholarship; discuss the scope of application/generalizability; identify unique contribution(s) to scholarship in a discipline or field.	Discussion; paragraphs 1–4<br>Discussion; Implications; paragraphs 1–4<br>Discussion; Strengths and limitations; paragraphs 1 & 2<br>Discussion; Recommendations for future research; paragraphs 1 & 2<br>Discussion; Conclusions; paragraph 1
Limitations	Discuss the trustworthiness and limitations of findings	Discussion; Strengths and limitations; paragraph 2
**Other**		

Conflicts of interest	Describe any potential sources of influence or perceived influence on study conduct and conclusions. Describe how these were managed.	Title Page; competing interests statement
Funding	Describe sources of funding and other support. Describe the role of funders in data collection, interpretation, and reporting.	Title Page; funding statement

1 How to specify where content is

Tell the reader where they can find information. E.g.,

- Results; paragraph 2
- Methods, Participants; paragraphs 1 & 2.
- Table 3
- Supplement B, para. 4

If you have chosen not to describe an item, explain why. You can do this in the checklist, or as a note below it.

You can describe items in the article body, or in tables, figures, or supplementary materials, and should prioritize items you feel are most important to your intended audience. The order of items in your manuscript does not need to match the order of items in this checklist. You can decide how best to structure your work.

2 How to cite

Describe how you used SRQR at the end of your Methods section, referencing the resources you used e.g.,

> 'We used the SRQR reporting guideline(1) to draft this manuscript, and the SRQR reporting checklist(2) when editing, included in supplement A'

If you use a reporting checklist, remember to include it as a supplement when publishing so that readers can easily find information and see how you have interpreted the guidance.

1. O'Brien BC, Harris IB, Beckman TJ, Reed DA, Cook DA. Standards for reporting qualitative research: A synthesis of recommendations. Academic Medicine [Internet]. 2014 Sep;89(9):1245–51. Available from: https://journals.lww.com/academicmedicine/fulltext/2014/09000/Standards_for_Reporting_Qualitative_Research__A.21.aspx

2. O'Brien BC, Harris IB, Beckman TJ, Reed DA, Cook DA. The SRQR reporting checklist. In: Harwood J, Albury C, Beyer J de, Schlüssel M, Collins G, editors. The EQUATOR network reporting guideline platform [Internet]. The UK EQUATOR Centre; 2025. Available from: https:/resources.equator-network.org/reporting-guidelines/srqr/srqr-checklist.docx
