# Supplementary material for: Facilitators and barriers for the implementation of intersectoral collaboration for health among Dutch primary and community care professionals: a qualitative study
Source: BMJ Public Health. 2026 Nov 6;4(4):e004909. doi: 10.1136/bmjph-2026-004909 (PMC13756415; doi:10.1136/bmjph-2026-004909)
Supplement: Supplementary file 2 [file bmjph-4-4-s002.pdf]

Appendix 1

Interview guide

Materials

Interview guide

Consent form

Print-out of the Positive Health spider web

Voice recorder

Notebook and pen

Introduction

Thank you very much for taking the time to participate in this interview today.

→ Does the participant prefer informal or formal address?

→ Does the participant consent to audio recording of the interview?

My name is [interviewer name]. I work at [educational institution] and together with several colleagues, we want to improve healthcare. Topics of interest include collaboration between professionals in healthcare, lifestyle, and Positive Health. We are developing educational materials that support healthcare professionals like yourself in applying these practices. If you have not heard of Positive Health before, that is no problem, I can explain it briefly later on.

Today, I would like to explore your experiences in collaborating with other professionals in healthcare, as well as applying lifestyle and Positive Health in your work. We will talk about this for approximately 30 to 60 minutes. You may withdraw from this study at any time, without providing a reason. I would like to ask that you answer as honestly as possible, so that we can better align our educational programme with real-world practice.

Do you have any questions you would like to ask before we begin?

Could you briefly tell me a bit about yourself?
E.g. age, profession, workplace, educational background, years of experience

Interdisciplinary Collaboration

Do you collaborate with other care professionals in your work?

→ If yes: How often does this happen?

Which care professionals are typically involved?

How does this collaboration occur? Who contacts whom?

→ If no: Why not?

What reasons do you see to collaborate with other care professionals?

Can you think of any reasons <u>not</u> to engage in such collaboration?

Lifestyle and prevention

Preventing illness can take place at several levels (see Figure).

Figure: Prevention pyramid

At this point in the interview, participants were shown the prevention period as presented in "Preventie: Wat is het en hoe benutten we het optimaal?" [Prevention: What is it and how do we make optimal use of it?).[1] The figure is not reproduced here; readers are referred to the original publication.

What does prevention mean to you?

What role does prevention play in the work that you do?

→ Which forms of prevention appear in your work?

Lifestyle counselling can be considered a form of prevention.

→ What reasons do you see for applying lifestyle counselling in your work?

→ Can you think of any reasons <u>not</u> to apply lifestyle counselling?

Positive Health

Have you heard of Positive Health before?

→ If yes: How did you come to know Positive Health?

→ If no: Provide the explanation below and discuss the spider web.

"Positive Health is a broader view of health. Instead of defining health as the absence of complaints or medical problems, more and more people agree that being healthy means being able to adapt to what happens in life. Based on research into what people themselves consider important for their health, six dimensions of health were identified. We are

interested in understanding how these different dimensions of health appear in healthcare practice."

What does Positive Health mean to you?

What role does Positive Health play in the work that you do?

→ Which dimensions of Positive Health appear in your daily practice?

→ What reasons do you see for applying Positive Health in your work?

→ Can you think of any reasons not to apply Positive Health in your work?

Closing

Is there anything we have not discussed during the interview that you would still like to mention?

References

1. Stronks K, Burdorf A, Mölenberg FJM, Kiefte JC, Kremers S, Assendelft WJJ, Mierau JO, Peters LL, de Wit NJ, van der Ven GL, Ballak DB. Preventie: wat is het, en hoe benutten we het optimaal? [Prevention: what is it, and how do we make optimal use of it?] [Internet]. 2022 [cited 2026 Sep 16]. Available from https://www.nfu.nl/sites/default/files/2022-07/22.01619_NFU_paper_preventie%27wat%20_is_het_en_hoe_benutten_we_het_optimaal%27.pdf
